# Establishment and characterization of a novel primary hepatocellular carcinoma cell line with metastatic ability *in vivo*

**DOI:** 10.1186/s12935-014-0103-y

**Published:** 2014-10-09

**Authors:** Phyllis Fung-Yi Cheung, Chi Wai Yip, Linda Wing-Chi Ng, Kwok Wai Lo, Nathalie Wong, Kwong Wai Choy, Chit Chow, Kui Fat Chan, Tan To Cheung, Ronnie Tung-Ping Poon, Sheung Tat Fan, Siu Tim Cheung

**Affiliations:** Department of Surgery, The University of Hong Kong, Hong Kong, China; Center for Cancer Research, The University of Hong Kong, Hong Kong, China; State Key Laboratory for Liver Research, The University of Hong Kong, Hong Kong, China; Department of Anatomical and Cellular Pathology, The Chinese University of Hong Kong, Hong Kong, China; Department of Obstetrics and Gynaecology, The Chinese University of Hong Kong, Hong Kong, China; Department of Pathology, Tuen Mun Hospital, Hong Kong, China

**Keywords:** Hepatocellular carcinoma, Cell line establishment, Granulin-epithelin precursor

## Abstract

**Background:**

Hepatocellular carcinoma (HCC) is a highly aggressive and heterogeneous disease. HCC cell lines established from different patients would be useful in elucidating the molecular pathogenesis. However, success of HCC primary culture establishment remains at low rate. We aim to establish and characterize HCC primary culture and the derived cell line.

**Methods:**

Fresh tumor tissues were collected from 30 HCC patients. Culture conditions were optimized for the attachment and growth of the isolated hepatocytes. Granulin-epithelin precursor (GEP), a growth factor reported to associate with cancer stem cell properties, was examined by flow cytometry to elucidate its role on primary culture establishment. The primary cell line was characterized in detail.

**Results:**

Cells isolated from 16 out of 30 HCC cases (53%) had viability more than 70% and were subject to subsequent *in vitro* culture. 7 out of 16 cases (44%) could give rise to cells that were able to attach and grow in culture. GEP expression levels significantly correlated with the viability of isolated hepatocytes and success rate of subsequent primary culture establishment. Cells from HCC patient 21 grew and expanded rapidly *in vitro* and was selected to be further characterized. The line, designated HCC21, derived from a Hong Kong Chinese female patient with HCC at Stage II. The cells exhibited typical epithelial morphology and expressed albumin, AFP and HBV antigens. The cell line was authenticated by short tandem repeat analysis. Comparative genome hybridization analysis revealed chromosomal loss at 1p35-p36, 1q44, 2q11.2-q24.3, 2q37, 4q12-q13.3, 4q21.21-q35.2, 8p12-p23, 15q11.2-q14, 15q24-q26, 16p12.1-p13.3, 16q, 17p, 22q and gain at 1q21-q43 in both HCC21 cells and the original clinical tumor specimen. Sequence analysis revealed p53 gene mutation. Subcutaneous injection of HCC21 cells into immunodeficient mice showed that the cells were able to form tumors at the primary injection sites and metastatic tumors in the peritoneal cavity.

**Conclusions:**

The newly established cell line could serve as useful *in vitro* and *in vivo* models for studying primary HCC that possess metastasis ability.

**Electronic supplementary material:**

The online version of this article (doi:10.1186/s12935-014-0103-y) contains supplementary material, which is available to authorized users.

## Background

Hepatocellular carcinoma (HCC) is one of the most common and aggressive human malignancies worldwide [1]. It is a highly heterogeneous disease in terms of its multiple molecular profiles and varied clinical outcomes [1]. The heterogeneous nature of HCC has impeded patient prognosis and treatment. Establishment of cell lines derived from different HCC patients is therefore useful for comprehensive study of pathogenesis of the disease. However, cell line establishment from fresh tumor tissues is technically difficult. Pre-operative procedures such as embolization and chemotherapy often induce extensive necrosis in tumor tissues and only small amount of viable tumor cells are available for subsequent culture. Besides, contamination and subsequent out-growth of infiltrating fibroblasts also made the cell line establishment more difficult. Therefore, the success rate of cell line establishment from fresh tumor tissues is extremely low [2,3], and the situation is even worse for cell lines with metastatic potential [4].

Anoikis is another critical factor hindering cell line establishment from fresh tissues. Anoikis refers to the apoptosis in epithelial cells due to the loss of interaction with their extracellular matrix. During hepatocyte isolation, cells were detached from extracellular matrix. Such detachment was shown to induce apoptosis of the freshly isolated cells and greatly decreased the cell viability and success rate of subsequent primary culture [5]. Hepatocyte isolation involves the use of collagenase degrading collagen and other intercellular material that support the structure of liver [6], which could trigger anoikis in freshly isolated hepatocytes [7,8]. Therefore, anoikis resistance is an important property that enables the freshly isolated cells to survive in the primary culture.

We previously demonstrated the granulin-epithelin precursor (GEP) was a hepatic oncofetal protein that defined a subpopulation of cancer stem cells (CSCs) in HCC [9]. It was proposed that CSCs possess certain properties that allow them to survive better than the other cell populations during primary culture [10]. However, the underlying mechanism was not well-understood. It was previously reported that GEP conferred anchorage-independence to cancer cells and protected them against anoikis [11,12]. Therefore, GEP-expressing cells might have advantage to survive better during hepatocyte isolation through resistance to anoikis.

Cell lines are used extensively in biomedical research as *in vitro* models for the cell type being investigated. Validity of the data obtained from cell lines depends on their identities, and how closely they resemble the characteristics of corresponding original tumor. For cell line identity, it is revealed that the frequency of cell line misidentification is high. Recent studies showed that between 18 and 36% of cell lines were incorrectly designated [13,14]. Accurate identification of cell lines is crucial during cell line development to avoid the risks of using misidentified cells. Short tandem repeat (STR) profiling has been recommended by the American Type Culture Collection Standards Development Organization (ATCC SDO) Workgroup ASN-0002 as the best method currently available for human cell line authentication [14,15]. For resemblance with original tumors, cell lines have been criticized for their inherent instability upon long term culture. In addition, culture process may lead to selective growth of rapidly growing cells that have more molecular abnormalities. Therefore, it is suggested that routine cell line authentication back to the original tissues is needed to ensure that the cell lines are still representative of the tumors. However, donor tissues are not available for most of the established cell lines, making such authentication impossible. HCC cell line in the early passages therefore provides a better experimental model for studying hepatocarcinogenesis as it resembles more closely the original tumor.

In present study, we aimed to establish new HCC cell line from fresh tumor tissues and optimize the culture conditions to facilitate the cell line establishment. We attempted to investigate the role of GEP in the viability of the freshly isolated cells and the success rate of subsequent primary culture. Here, we showed that GEP level was positively correlated with the viability of freshly isolated hepatocytes and the success rate of subsequent primary culture. The culture conditions for the primary hepatocytes were optimized and a new cell line, designated HCC21, was established from the fresh tumor tissue of a Hong Kong female patient with early staged and moderately differentiated HCC. The line was authenticated, and its morphology, growth kinetics, migration ability, cytogenetic features, and *in vivo* tumorigenicity were characterized. This newly established cell line should serve as a useful model for studying the molecular pathogenesis of HCC.

## Results

### Primary culture establishment from fresh tumor tissues of 30 HCC patients

**Figure 1:**
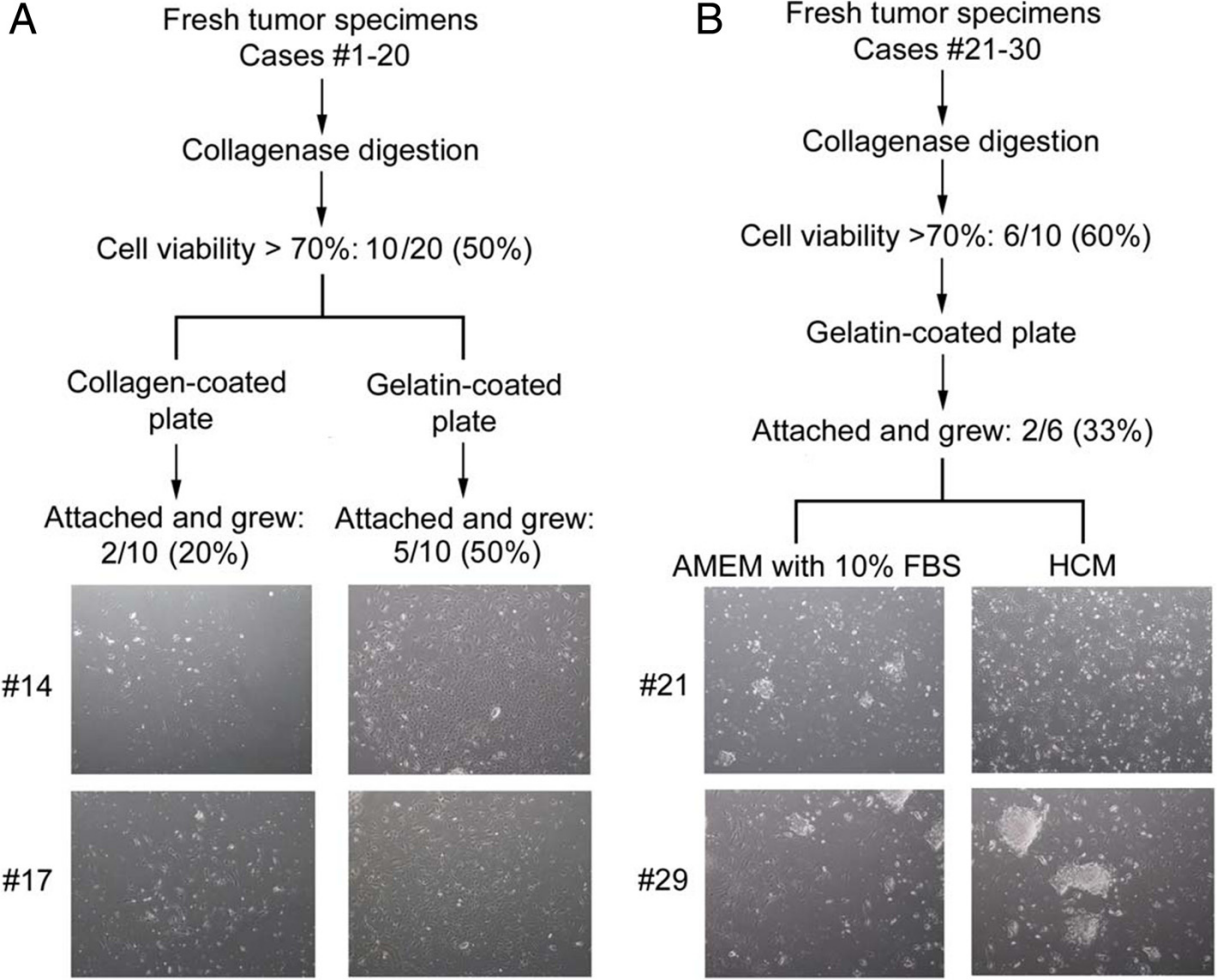
**Primary culture establishment from fresh tumor tissues of 30 HCC patients.** Experimental protocol for establishing primary HCC culture from 30 patients was illustrated. Fresh tumor tissues were collected from HCC patients and underwent enzymatic digestion by type IV collagenase to release disaggregated cells. **(A)** For cases #1-20, cells were resuspended in AMEM supplemented with 10% FBS and then seeded onto 6-well culture plates coated with either 0.1% gelatin or 0.1% collagen. Phase contrast microscopy images of cultured cells from cases #14 and #17 were shown. **(B)** For cases #21-30, cells were resuspended in either AMEM supplemented with 10% FBS or HCM and then seeded onto 6-well culture plates coated with 0.1% gelatin. Phase contrast microscopy images of cultured cells from cases #21 and #29 were shown.

Fresh tumor tissues from 30 HCC patients were included in the primary culture establishment study. After enzymatic digestion by type IV collagenase, disaggregated cells were collected from the tumors. Cell viabilities were assessed and only cases with cell viability >70% were subject to subsequent *in vitro* culture. For cases #1 to #20, 10 out of 20 cases (50%) generated cells with >70% viability. Primary cells usually require extracellular matrix components such as collagen and fibronectin or biodegradable polymers such as gelatin to promote cell attachment. Of these, gelatin and collagen were reported to favour sustained viability and functions of hepatocytes [16]. Therefore, freshly isolated cells were resuspended in AMEM supplemented with 10% FBS and then seeded onto 6-well culture plates coated with either gelatin or collagen. Cells from 5 out of 10 cases (50%) attached to the culture plates coated with 0.1% gelatin; while only 2 out of 10 cases (20%) attached to those coated with 0.1% collagen. Cells growing on collagen-coated plate were sparse and die within 1 month, while those growing on gelatin-coated plate attached better and could establish cell-to-cell contacts (Figure 1A). Therefore, 0.1% gelatin was chosen to coat plate for primary cell culture of subsequent HCC cases.

Fibroblast contamination and subsequent out-growth was frequently found in the process of cell line establishment. Therefore, we started to use defined media, hepatocyte culture medium (HCM), for cases #21 to #30. HCM provides a condition that permits preferential growth of hepatocytes, while that of contaminating fibroblasts is not favoured. For cases #21 to #30, 6 out of 10 cases (60%) generated cells with >70% viability, and the cells were resuspended in either AMEM supplemented with 10% FBS or HCM and then seeded onto 6-well culture plates coated with gelatin. Among the 6 cases, cells of #21 and #29 (2 out of 6 cases, 33%) could attach to culture plates coated with gelatin and grow in both 10% AMEM and HCM (Figure 1B). Fibroblast out-growth was observed in both cases cultured in AMEM with 10% FBS, while fibroblasts gradually decreased and disappeared in those cultured in HCM. Cells from #21 grew well and could propagate in subsequent passages, while those from #29 died gradually after one passage. Extensive characterization of cells from #21 was then performed.

### Correlation of GEP expression with success rate of HCC primary culture

We previously demonstrated that GEP was a hepatic oncofetal protein that defined CSC population in HCC [9]. CSCs are proposed to possess certain properties that allow them to survive better in primary culture [10]. Further analysis with clinico-pathological features revealed that increased GEP level was significantly associated with poor differentiation (p = 0.044) (Additional file 1: Table S1). As tumors with poor differentiation were more aggressive and associated with more stem-cell features [17], the result implied that GEP might contribute to the aggressiveness and stemness of HCC, and GEP-expressing cells might survive better in primary culture.

**Figure 2:**
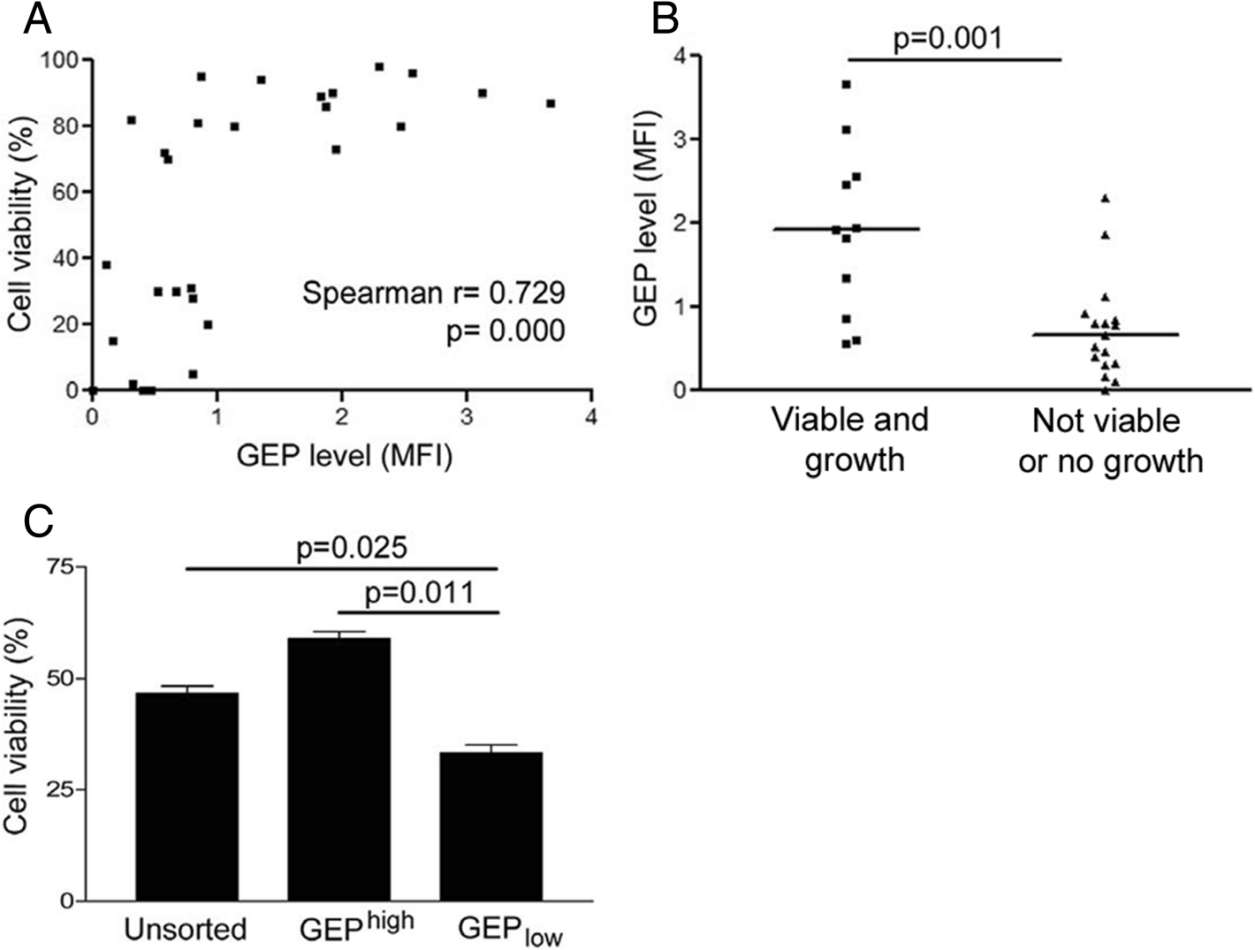
**Correlation of GEP expression with success rate of HCC primary culture. (A)** GEP levels significantly correlated with the viability of the cells freshly isolated from HCC tissues (n = 28, Spearman’s ρ correlation coefficient = 0.729, p = 0.000). GEP level was measured by flow cytometry and expressed as mean fluorescence intensity (MFI) after subtracting the non-specific background signal (isotype control). **(B)** GEP levels (MFI) of the cells with viability > 70% and could grew on culture plate successfully (viable and growth) were significantly higher than those failed or viability <70% (not viable or no growth) (n = 28, p = 0.001). **(C)** Hep3B cells were sorted for surface GEP expression by magnetic sorting. Unsorted control cells, sorted GEP^high^ and GEP_low_ cells were then cultured in 10% FBS-supplemented AMEM in ultra-low attachment plate for 12 h. Cells were harvested and stained for annexin V-propidium iodide for apoptosis. Cells negative for both annexin V and propidium iodide were defined as viable cells, which were quantified by flow cytometric analysis. Viability of GEP_low_ cells was significantly lower than unsorted and sorted GEP^high^ cells.

We quantified the GEP levels of the original tumors by flow cytometry, and studied their relationship with the cell viability and success rate of cell line establishment. GEP level was shown to be significantly correlated with the viability of cells isolated from the fresh tumor tissues (n = 28, Spearman’s ρ correlation coefficient = 0.729, *p =*0.000) (Figure 2A). Cells isolated from 7 cases showed viability >70% and could successfully grow in culture, and their GEP levels were significantly higher than those that failed to grow or the viability was <70% (p = 0.001, Figure 2B). The results suggested that GEP might be an important factor for facilitating cell line establishment from fresh HCC tumor tissues.

During hepatocyte isolation, cells were detached from extracellular matrix. Such detachment induced anoikis and greatly decreases the cell viability and success rate of subsequent primary culture [5]. GEP was shown to protect cancer cells against anoikis [11,12], and might therefore facilitate primary culture establishment by conferring anoikis resistance to the HCC cells. To support this hypothesis, Hep3B cells were sorted based on GEP expression. Post-sorting analysis was performed and purity was shown to be >80% (Additional file 1: Figure S1). Unsorted control cells, sorted GEP^high^ and GEP_low_ cells were then cultured in attachment free environment for 12 h. Viability of GEP_low_ cells was significantly lower than unsorted and sorted GEP^high^ cells (p = 0.025 and p = 0.011, respectively), suggesting that GEP was crucial for protecting HCC cells from anoikis-induced apoptosis (Figure 2C) and might facilitate primary culture establishment.

### Phenotypic characterization of HCC21 cells

**Figure 3:**
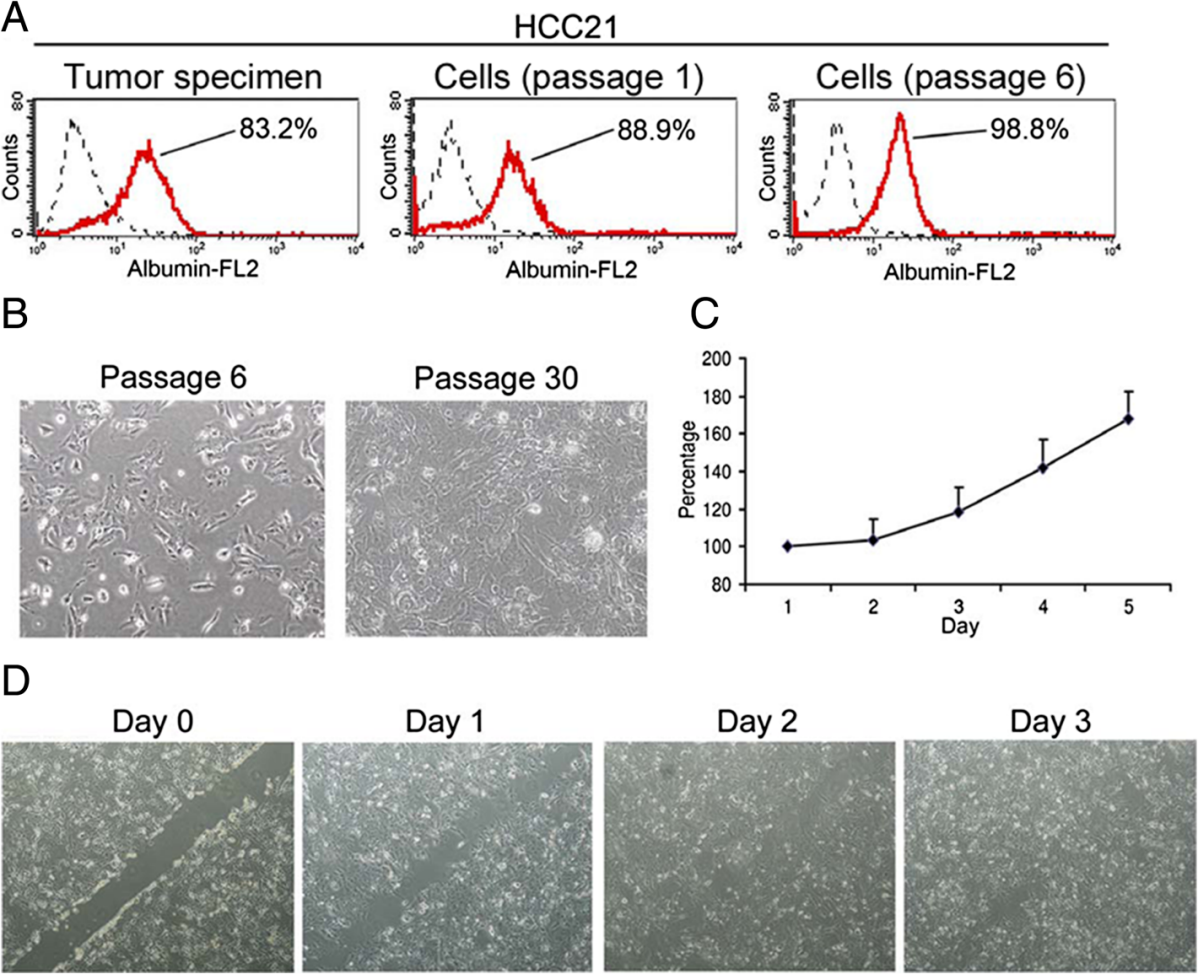
**Phenotypic characterization of HCC21 cells. (A)** Albumin levels of freshly isolated cells from patient’s tumor specimen, HCC21 cells at passage 1 and 6 were measured by flow cytometry. Percentages of albumin + cells were indicated in the histograms. **(B)** Phase-contrast microscopy images of cultured HCC21 cells at passages 6 and 30. **(C)** Growth curve of HCC21 cells at passage 10. **(D)** Wound healing assay showing the migration ability of HCC21 cells at passage 10.

The cells derived from HCC case #21, designated HCC21, reached 80% confluency at 10 days after initial culture, and sub-passage was performed successfully without significant cell death. The cells began to grow quickly at the 6th passage and were passaged for more than 50 generations thereafter. Flow cytometric analysis showed that albumin + cells of the original tumor #21 were 83.2%. Upon culture for about 10 days in hepatocyte culture medium, albumin + cells increased to 88.9%, which was further enriched to more than 98% after 6 passages (Figure 3A), confirming that HCC21 cells were hepatocytes. HCC21 cells grew as adherent monolayer with epithelial morphology (Figure 3B) and the cells maintained consistent morphology along passages. Growth curve of HCC21 cells is shown in Figure 3C. The population doubling time of HCC21 was approximately 95 h. Wound healing assay was performed to assess the migration ability of HCC21 cells. The cells started to migrate a day after the wound was made, and the wound healing was completed after 3 days (Figure 3D). The migration ability of HCC21 cells echoed the histology observation that venous infiltration (micrometastasis) was observed in the surgical specimen.

### STR profile analysis of HCC21 cells

**Figure 4:**
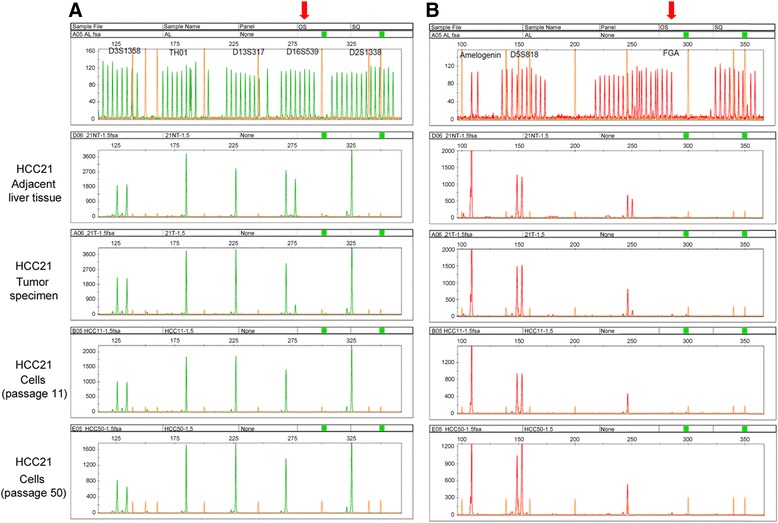
**DNA fingerprinting of HCC21 cells.** Comparison of genotyping results in adjacent non-tumor liver tissue and tumor specimen from patient, and HCC21 cells at passage 11 and 50. Patient’s tumor specimen and cultured HCC21 cells demonstrated loss of heterozygosity in **(A)** D16S539 and **(B)** FGA when compared to adjacent non-tumor liver tissue.

The DNA samples of HCC21 early and late passages (11 and 50, respectively), original tumor and adjacent non-tumor liver tissue were subjected to DNA fingerprinting analysis. A total of 15 STR loci (CSF1P0, D2S1338, D3S1358, D5S818, D7S820, D8S1179, D13S317, D16S539, D18S51, D19S433, D21S11, FGA, TH01, TPOX, vWA) were co-amplified in each sample. *AMLEO* locus at the sex chromosomes was also examined. The data were analyzed and allele(s) of each locus were determined (Table 1). The STR profiles of HCC21 cells and the original tumor were identical, suggesting that they came from the same person. Besides, loss of heterozygosity (LOH) was observed in 2 loci, D16S539 and FGA in original tumor, and the aberrations were retained in HCC21 cells (Figure 4).

**Table 1:** **STR profiles of HCC21 cells at passage 11 and 50, adjacent non-tumor liver tissue and tumor specimen from patient**

	**HCC21**	**HCC21**	**HCC21**	**HCC21**
	**Cells (passage 11)**	**Cells (passage 50)**	**Adjacent liver tissue**	**Tumor specimen**
D8S1179	14	14	14	14
D21S11	30, 31.2	30, 31.2	30, 31.2	30, 31.2
D7S820	10, 11	10, 11	10, 11	10, 11
CSF1PO	11	11	11	11
D3S1358	15, 17	15, 17	15, 17	15, 17
TH01	9	9	9	9
D13S317	10	10	10	10
D16S539	9	9	9, 11	9, 11 (LOH)
D2S1338	19	19	19	19
D19S433	13.2, 14.2	13.2, 14.2	13.2, 14.2	13.2, 14.2
vWA	16, 17	16, 17	16, 17	16, 17
TPOX	8, 12	8, 12	8, 12	8, 12
D18S51	12, 15	12, 15	12, 15	12, 15
Amelogenin	X	X	X	X
D5S818	10, 11	10, 11	10, 11	10, 11
FGA	24	24	24, 25	24, 25 (LOH)

### Array-based comparative genomic hybridization (aCGH) analysis

**Figure 5:**
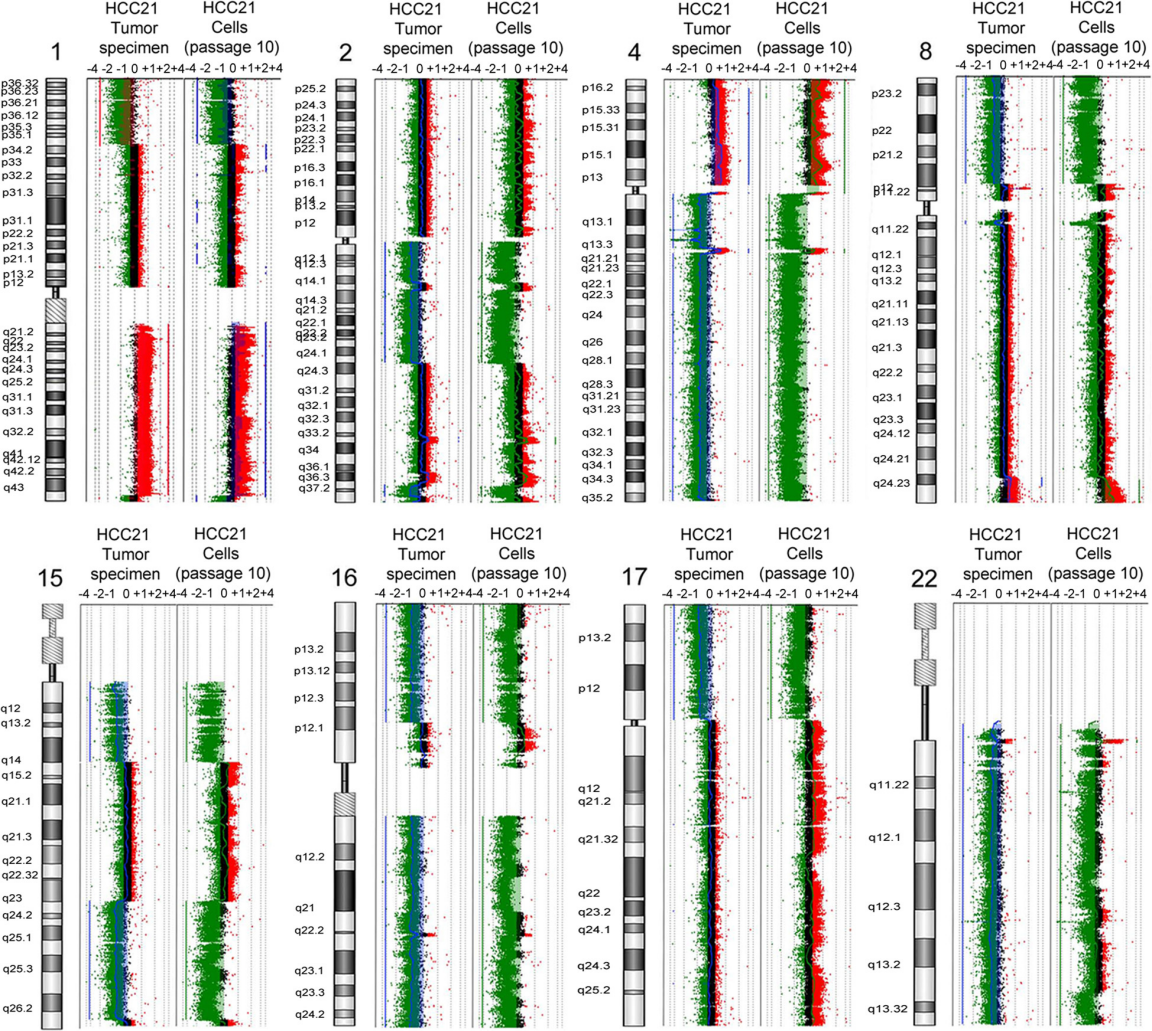
**CGH analysis of HCC21 cells and the original tumor.** Vertical profiles of chromosomes 1, 2, 4, 8, 15, 16, 17 and 22 are shown with a log_2_ ratio, which represents the difference between tumor and normal DNA copy number. Ratio of 0 indicates that there is no change in copy number between tumor and normal cell DNA. Positive and negative log_2_ ratios indicate the gain and loss of tumor DNA copy number, respectively. The left panel of each plot represents the chromosome of the original clinical tumor specimen, while the right panel represents that of HCC21 cells at passage 10.

Changes in genomic copy number in HCC21 cells and original tumor were characterized by aCGH analysis. aCGH arrays were performed using DNA from original tumor and that from the HCC21 cells 6 months after its establishment. Chromosomal aberrations including chromosomal loss at 1p35-p36, 1q44, 2q11.2-q24.3, 2q37, 4q12-q13.3, 4q21.21-q35.2, 8p12-p23, 15q11.2-q14, 15q24-q26, 16p12.1-p13.3, 16q, 17p, 22q and gain at 1q21-q43 were observed in both the original tumor specimen and HCC21 cells (Figure 5). Noted the STR profiles showed LOH at FGA (at chromosome 4q28) and D16S539 (at chromosome 16q24.1), and that corroborated the aCGH data with chromosomal loss at 4q21.21-q35.2 and 16q in both the original clinical tumor specimen and the derived HCC21 cells.

### TP53 mutational analysis

**Figure 6:**
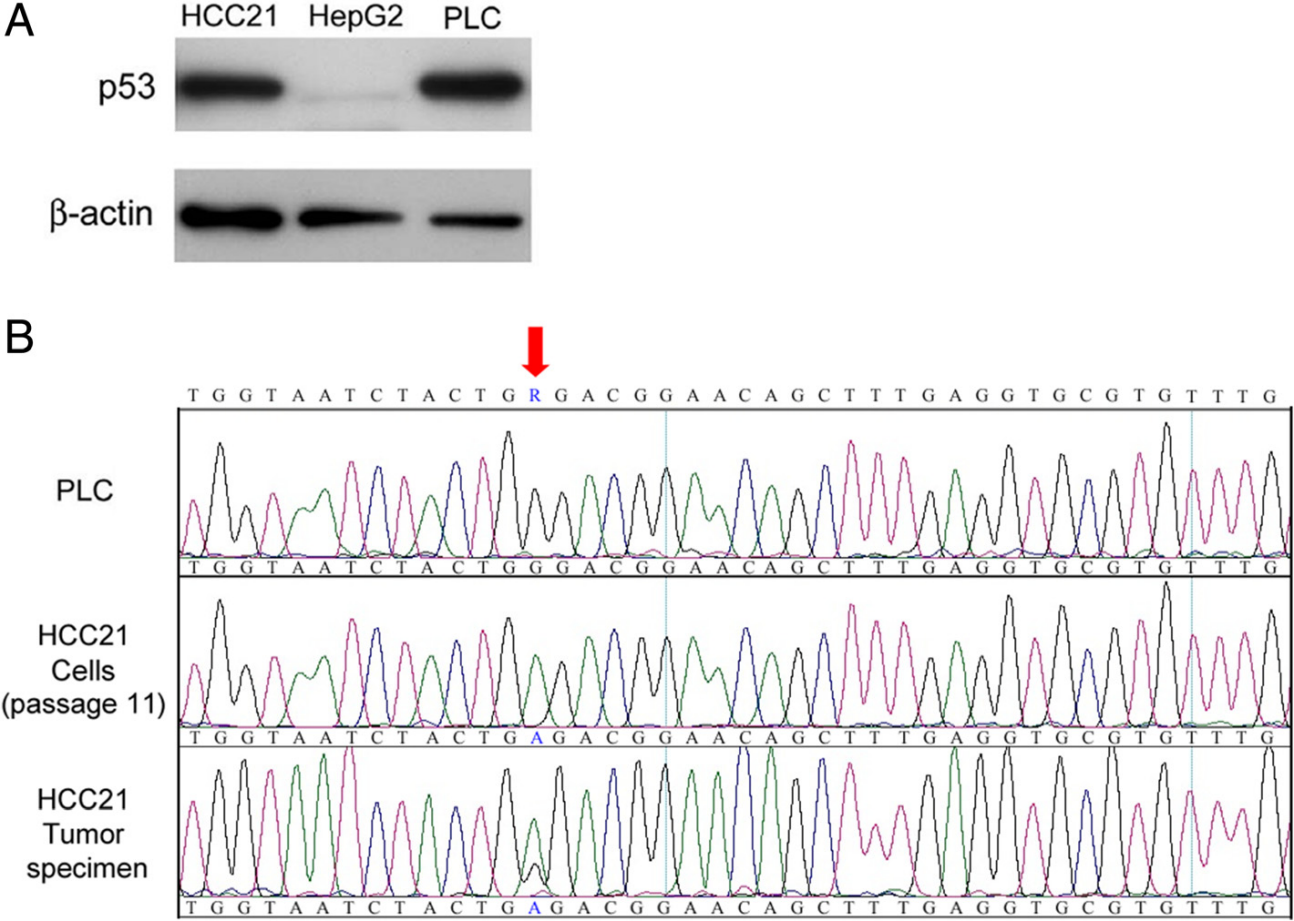
**TP53 mutational analysis in HCC21 cells. (A)** Expression of p53 protein in HCC21 cells, HepG2 (wild-type p53) and PLC (p53 mutation at codon 249). **(B)** Sequencing analysis of TP53 gene revealed a point mutation (Gly→Arg) in codon 266 of exon 8 in HCC21 cells (passage 11) and patient’s tumor specimen, but not PLC.

p53 has been reported to be frequently mutated and overexpressed in HCCs [18]. Result from western blot showed that p53 protein was present in HCC21 cells. Human liver cancer cell lines HepG2 and PLC/PRF/5 were included as wild-type and mutant controls, respectively (Figure 6A). Subsequent analysis by DNA sequencing indicated a point mutation (Gly → Arg) (GGA → AGA) at codon 266 of exon 8 in both the HCC21 cells and tumor specimen (Figure 6B). PLC/PRF/5 was included as p53 mutant control (codon 249 of exon 7) and no mutation was found in exon 7 of HCC21 cells (data not shown).

### Tumorigenicity and metastatic potential in immunodeficient mice

**Figure 7:**
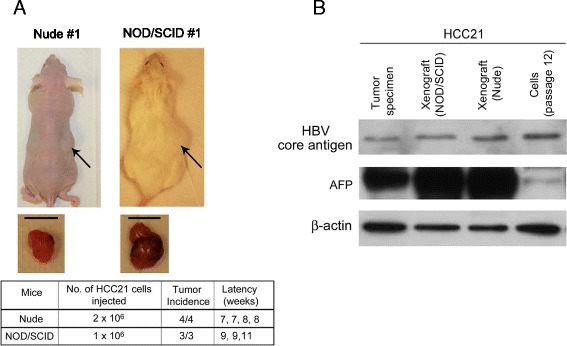
**Tumorigenicity of HCC21 cells in immunocompromised mice. (A)** Nude mice and NOD/SCID mice injected subcutaneously with 2x10^6^ and 1x10^6^ HCC21 cells, respectively, after 12 weeks. The middle panel shows the subcutaneous tumors derived from HCC21 cells 12 weeks after injection (scale bar = 10 mm). The lower panel shows the tumorgenicity of HCC21 cells. **(B)** Protein expression of HBV core antigen and AFP in patient’s tumor specimen, HCC21 xenograft tumors in NOD/SCID and nude mice, and cultured HCC21 cells at passage 12.

**Figure 8:**
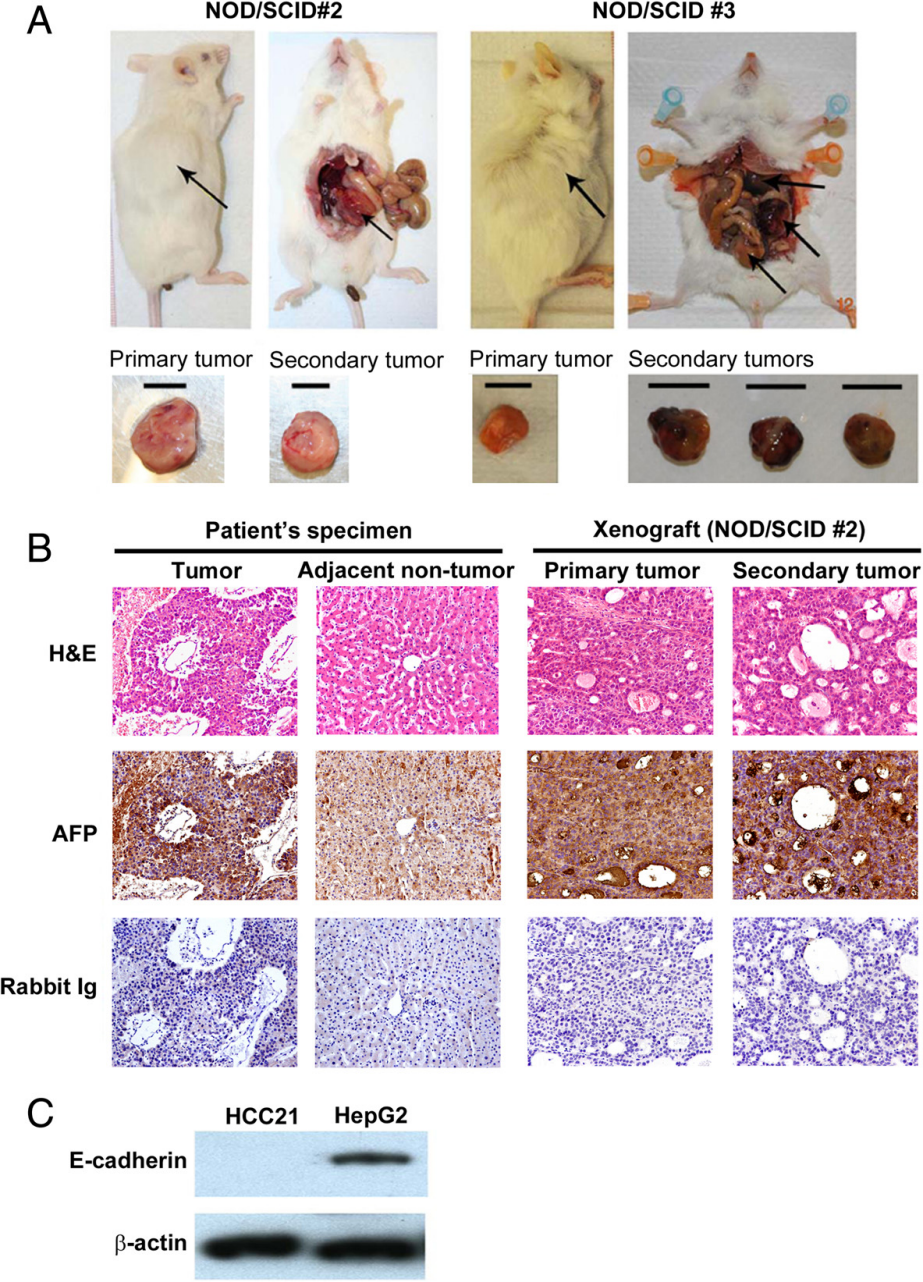
**Metastatic potential of HCC21 cells in NOD/SCID mice. (A)** Subcutaneous injection of HCC21 cells into NOD/SCID mice led to primary tumor development at the site of injection and secondary tumors in the peritoneal cavity (scale bar = 10 mm). **(B)** Histological analysis of patient’s tumor and adjacent non-tumor liver tissues, primary and secondary xenograft tumors established in NOD/SCID mice. Sections were stained with haematoxylin and eosin (H&E) (upper panel), AFP (middle panel) and rabbit Ig control (lower panel). **(C)** Undetectable protein expression of E-cadherin in HCC21 (passage 12) while HepG2 served as positive control.

HCC21 cells were injected subcutaneously into 4 nude and 3 NOD/SCID mice. About 2 months after injection, visible tumors developed in all mice at the site of inoculation (Figure 7A), indicating that HCC21 cells were tumorigenic. We assessed the expression of HCC markers including AFP and HBV antigen [19] in the HCC21 cells and xenograft tumors. Western blot analysis showed that HCC21 cells and xenograft tumors in both nude and NOD/SCID mice retained the expression of HBV core antigen and AFP, which was consistent with original resected tumor (Figure 7B). Most importantly, secondary tumors were found in the peritoneal cavities of 2 NOD/SCID mice (Figure 8A). Histological sections of the xenografts showed neoplastic hepatocytes proliferation in broad trabeculae and acinar pattern, morphologically consistent with HCC. In addition, the metastatic (secondary) lesions were morphologically similar to the primary tumors (Figure 8B, upper panel). Besides, we have examined the expression of AFP in the primary and secondary xenograft tumors, in comparison to the patient’s tumor and adjacent non-tumor liver tissue specimen by immunohistochemical staining. Results showed that AFP protein was expressed at high levels in the tumor specimen, and also in the primary and secondary xenograft tumors. AFP protein could also be detected in adjacent non-tumor liver tissue specimen, but the signal was lower than the tumor counterpart (Figure 8B, middle panels). This further demonstrated that the tumor characteristics had been preserved from the original tumor specimen to the primary and metastasized tumor xenografts.

The aCGH analysis revealed loss of 16q in HCC21 cells, which region contained E-cadherin gene (at chromosome 16q22.1). E-cadherin is an adhesion molecule crucial for inhibiting metastasis by connecting homophilic cells [20-22]. Loss of E-cadherin was reported to associate with high incidence of lymph node metastasis in various cancers, including HCC [23-25]. Therefore, we examined the expression of E-cadherin in HCC21 cells by western blot. HepG2, HCC cell line without metastatic potential, was included as positive control for the presence of E-cadherin. Result showed that E-cadherin protein was absent in HCC21 cells (Figure 8C), thereby providing further evidence for the metastatic potential of the cells.

## Discussion

HCC is a highly heterogeneous in terms of molecular profiles and clinical outcomes [1]. Establishment of primary HCC culture derived from different patients is therefore useful for comprehensive study of the etiology and molecular pathogenesis of disease. However, establishment of human cell lines from fresh patients’ tumors is technically difficult, partly due to the low viability of the freshly isolated HCC cells. Here, we showed for the first time that GEP protein levels of HCC cells were significantly correlated to the cell viability and also the success rate of subsequent primary culture. GEP-expressing cells were able to survive better during hepatocyte isolation, which was potentially due to their resistance to anoikis. The findings therefore suggested that GEP might be an important factor for facilitating cell line establishment from fresh HCC tumor tissues.

Recent reports on GEP binding partners might provide some hints on the underlying mechanism for GEP-mediated anoikis resistance. GEP was demonstrated as a ligand of tumor necrosis factor receptors (TNFRs) in human chondrocytes and disturbed TNFα signaling [26]. Notably, TNFR1 has been shown to be crucial on anoikis execution in cancer progression [27]. In addition, our group have recently demonstrated that GEP interacted with heparan sulfate on HCC cell surface [28]. Heparan sulfate was essential for anoikis resistance and survival in mouse dermal fibroblasts [29] and in rat embryo fibroblasts [30]. We have further shown that tropomyosin 3 was the intracellular binding partner of GEP in liver cancer cells [31]. Independently, tropomyosin-1 was reported to regulate anoikis in breast cancer cells [32]. Therefore, further investigations are warranted to investigate the roles of TNFR, heparan sulfate and tropomyosins on GEP-mediated anoikis resistance in human cancer cells.

Cell line authentication is crucial during cell line development to avoid the risks of using misidentified cells. Recent studies have shown that more than 360 cell lines were cross-contaminated or misidentified without authenticated stock, and the validity of the studies using these cell lines was in doubt [33]. A cell line is considered misidentified when its DNA profile is not consistent with the individual from whom it was derived from. Here, we showed that the STR profiles of HCC21 cells and original resected tumor were identical, confirming that HCC21 cells and the tumor came from the same person.

Chromosomal alterations in HCC are complex [34], and this has impeded the elucidation of molecular mechanisms underlying hepatocarcinogenesis. Chromosomal aberrations in HCC21 cells were examined using CGH. The results showed that chromosomal aberrations were observed in both the primary tumor and HCC21 derived cells including loss at 1p35-p36, 1q44, 2q11.2-q24.3, 2q37, 4q12-q13.3, 4q21.21-q35.2, 8p12-p23, 15q11.2-q14, 15q24-q26, 16p12.1-p13.3, 16q, 17p, 22q and gain at 1q21-q43. Of these, gain at 1q was previously reported to be associated with HCC development [35-37]. Loss of 4q11-23 was previously reported to be observed in tumor larger than 3 cm. This is consistent with the tumor size of patient (8.5 cm). Chromosome 4q abnormality was significantly associated with HCC [38,39]. LOH at 4q was reported to strongly correlate with AFP elevation in HCC [40], and this echoes the high serum AFP level (30690 ng/ml) in the patient. Loss of 17p was frequently found in HCC, which was generally explained by loss of the TP53 gene [41-43]. Loss of 8p was observed in both HCC21 cells and original tumor. This aberration was previously reported to significantly correlate with tumor metastasis in HCC.

Mutations in the TP53 tumor suppressor gene are found in approximately 50% of human cancers and the most common mutations are missense mutations leading to amino acid substitutions [43-45]. Since point mutation of genes can occur during serial *in vitro* passage, gene analysis of primary tumors is therefore indispensable. We showed here that a point mutation in the p53 suppressor gene (Gly → Arg at codon 266 at exon 8) was present in both HCC21 cells and the original tumor, thereby confirming the mutation was not induced during serial *in vitro* passage. This mutation has not been reported in HCC. Similar to other p53 mutation, this mutation may also result in biological altered protein with increased stability and nuclear accumulation in the cells [44]. This is supported by the high protein expression level of p53 in HCC21 cells, when compared with that in HepG2, the p53 wild type control cell line. However, further investigation is needed to elucidate the detailed mechanism.

HCC has been reported with high incidence of metastasis [46]. The underlying mechanism of metastasis is still poorly elucidated, which is probably due to the lack of good metastatic HCC cell line models for the related studies. Although a number of human HCC cell lines are currently available, few have demonstrated prominent metastatic potential [47,48]. To date, MHCC97 is the only well-characterized HCC cell line with metastatic potential [47]. However, it was established from subcutaneous xenograft of a metastatic model of human HCC in nude mice by means of alternating cell culture *in vitro* and growth in nude mice. Xenografts generated by implantation of cell lines were shown to have poor predictive power for translation of preclinical efficacy into clinical outcome [49]. HCC21, on the other hand, derived from fresh tumor tissue of HCC patient, might therefore serve as a better model for studying the molecular mechanism of metastasis in HCC.

Epithelial-mesenchymal transition (EMT) plays an important role in cancer progression and metastasis [50,51]. A hallmark of EMT is down-regulation of E-cadherin, a cell adhesion molecule essential for the establishment of stable adherent junctions. It has been reported that repression of E-cadherin is associated with dedifferentiation, infiltrative growth and high incidence of lymph node metastasis in several cancers, including HCC [23-25]. Here, CGH analysis showed that HCC21 cells exhibited loss of 16q21-24, which contained E-cadherin gene. E-cadherin protein was also confirmed to be absent in HCC21 cells, suggesting that the metastatic potential of the cells was at least partially due to the loss of E-cadherin. In addition, MMPs are also crucial for tumor metastasis by degrading and remodeling the extracellular matrix. A number of ECM degrading MMPs, such as MMP1, MMP2 and MMP14, have been implicated in the process of tumor invasion and metastasis [52]. Further investigation is needed to elucidate the role of these MMPs in the metastatic potential of HCC21 cells.

Most of the currently available cancer cell lines are derived from late-staged and poorly-differentiated tumors such that the cells have accumulated the mutations required for indefinite growth *in vitro*. However, cell lines from early-staged and well-differentiated tumors are needed for better understanding of the HCC progression and pathogenesis. The doubling time of HCC21 is 95 hours, which is relatively long when compared with other established HCC cell lines. The original tumor was classified as stage II based on TNM system and graded as moderately differentiated. Therefore, this cell line will serve as a relatively early-staged and moderately differentiated HCC model for biomedical research on early stage of hepatocarcinogenesis.

## Conclusions

In present study, a novel HCC cell line from a Hong Kong female patient with early-staged and moderately differentiated HCC was established and authenticated. This cell line reproduces the characteristics of the original tumor, and could provide researchers with a well-delineated and early-passage cell line model to investigate the biology and molecular details of HCC. Authentication of cell line is important to ensure that the cell line is representative of the original tumor, and serves as a relevant tool for research studies. In addition, we optimized the protocol for primary culture of HCC. The study also showed that GEP might be an important factor determining the viability of the freshly isolated HCC cells and therefore affect the success rate of subsequent primary culture. The study provided an improved protocol for establishment of primary culture of HCC, detailed characterization and authentication on the newly established cell line.

## Methods

### Specimen collection

The study protocol was approved by the Institutional Review Board of the University of Hong Kong / Hospital Authority Hong Kong West Cluster (HKU/HA HKW IRB). Between July 2010 and March 2011, 30 patients having curative partial hepatectomy or liver transplantation for HCC at Queen Mary Hospital, Hong Kong, were recruited after written informed consent was obtained. Tumors and non-tumorous tissues were collected from the resected specimens. A portion of tumor tissues was freshly processed for cell culture and flow analysis, while DNA and proteins were extracted from the snap frozen tissues.

Original tumor tissue from which HCC21 cells was derived was obtained from a 42-year-old Hong Kong Chinese female patient who underwent curative partial hepatectomy. Tumor size was 8.5 cm in diameter. The patient was seropositive for hepatitis B virus (HBV), and serum α-fetoprotein (AFP) level was 30690 ng/mL (reference range <10 ng/mL). A diagnosis of HCC arising in a cirrhotic liver was confirmed by histological examination. Venous infiltration was observed, indicating the aggressiveness of the tumor. The tumor was classified as stage II according to the pathological tumor-node-metastasis (pTNM) staging system 2009 version, and graded as moderately differentiated.

### Primary culture and cell line establishment

Fresh tumor tissues were rinsed with AMEM medium (Invitrogen, Carlsbad, CA) supplemented with 50 units/ml Penicillin G (Invitrogen) and 50 μg/ml streptomycin (Invitrogen), and then minced into 1-mm^3^ pieces. After digestion with type IV collagenase (Sigma, St. Louis, MO) for 5 min at 37°C for 3 times, disaggregated cell suspension was obtained by filtering through a 40 μm cell strainer (BD Biosciences, San Jose, CA). After lysis of red blood cells by ACK lysis buffer (Invitrogen), cells were washed with AMEM medium. The cells were then resuspended in either AMEM supplemented with 10% FBS (Invitrogen) or hepatocyte culture medium (Lonza, Basel, Switzerland), and then seeded onto 6-well culture plates coated with either 0.1% gelatin (Sigma) or 0.1% type I collagen (Sigma). Cells were incubated at 37°C in a humidified atmosphere at 95% air and 5% CO_2_ and left untouched for 2 days. The culture medium was then changed twice a week and cells were sub-passaged when they reached 70-80% confluency.

For HCC21 cells, a split ratio of 1:1 was applied in the early passages (passage 1 to 5), thereafter increased to 1:3. Cells were collected at different passages and put in freezing medium containing 50% hepatocyte culture medium, 40% FBS and 10% DMSO, and stored in liquid nitrogen. The cells were tested for mycoplasma contamination and the result was negative.

### Anoikis assay

Hep3B cells were sorted for GEP^high^ and GEP_low_ subpopulations by magnetic activated cell sorting (Miltenyi Biotec) as previously described [9]. After cell isolation, one million of each sorted population was collected to assess cell viability and purity by trypan blue staining and flow cytometry, respectively. Post-sorting analysis typically indicated purities of at least >80% with minimal cell death (<10%). Unsorted control cells, sorted GEP^high^ and GEP_low_ cells were then cultured in 10% FBS-supplemented AMEM in ultra-low attachment plate (Corning Inc, Corning, NY) for 12 h. Cells were harvested and stained for annexin V-propidium iodide (BD Biosciences) for viability, in which cells negative for both annexin V and propidium iodide were defined as viable cells. Viable cells were quantified by flow cytometric analysis.

### Immunofluorescence staining and flow cytometric analysis

Cells were permeabilized with ice-cold 0.1% saponin and then incubated with FITC-conjugated mouse anti-human GEP antibody (described previously [53]) (Versitech Ltd, Hong Kong), mouse anti-human albumin (R&D systems, Minneapolis, MN) or equal amount of mouse IgG isotype (Sigma). Cells were washed with 0.1% saponin and then subject to flow cytometric analysis. Results were expressed as percentage of positive cells or mean fluorescence intensity (MFI), after subtracting the non-specific background signal (isotype control).

### Morphological examination and growth kinetics

Cells were routinely monitored and photographed with a phase-contrast microscope. Cells of passages 6 and 16 were studied to measured the population doubling time, which was assessed by 3-(4,5-dimethylthiazol-2-yl)-2,5-diphenyltetrazolium bromide (MTT) assay for 5 consecutive days.

### Would healing assay

Cells were seeded onto a 6-well culture plate and incubated for 24 hr. A wound was made by scraping a 20 μl pipette tip across the cell monolayer. Cells were rinsed with PBS and cultured in HCM for 3 days. Cell movement toward the wound was observed under a phase-contrast microscope every 24 h after the wound was made.

### Short tandem repeat (STR) analysis

DNA samples of the HCC21 cells at passage 11 and 50, original tumor and the adjacent non-tumor liver tissue from the patient were subjected to DNA fingerprinting analysis using the AmpF/STR Identifiler Plus PCR Amplification Kit (Invitrogen). A total of 15 STR loci (CSF1P0, D2S1338, D3S1358, D5S818, D7S820, D8S1179, D13S317, D16S539, D18S51, D19S433, D21S11, FGA, TH01, TPOX, vWA) were co-amplified in each sample and detected on an ABI 3130 Genetic Analyzer. *AMLEO* locus at the sex chromosomes was also examined. The data were analyzed and allele(s) of each locus were determined by GeneScan and GeneMapperTM ID Software (Invitrogen).

### Array-based comparative genomic hybridization (aCGH)

Genomic DNA was extracted from HCC21 cells and the original tumor. The Agilent human 44 K CGH microarray (Agilent Technologies, Santa Clara, CA) with an average probe spatial resolution of ∼ 35 kb was utilized. Genomic DNA at 1 μg was labelled by the Agilent Genomic DNA Labeling Kit PLUS (Agilent Technologies). The hybridized array was scanned by an Agilent Microarray Scanner and data were analysed by Agilent Feature Extraction 9.1 followed by computation and normalization using CGH Analytics v3.4. Putative chromosome copy number loss was defined by intervals of two or more adjacent probes with log_2_ ratios suggestive of a deletion when compared with the log_2_ ratios of adjoining probes. The Quality-Weighted Interval Score algorithm (ADM2) with a value of 6.0 was used to compute and assist the identification of aberrations for a given sample.

### Western blot analysis

Total protein was extracted with cell lysis buffer (Cell Signaling Technology, Boston, MA) in the presence of complete protease inhibitor cocktail (Roche, Mannheim, Germany) and separated in 8-10% SDS PAGE gel. Proteins were then electro-transferred onto polyvinylidene difluoride membranes, subsequently incubated with primary anti-human antibodies, detection by horseradish peroxidase-labeled secondary antibodies, and visualized with Enhanced Chemiluminescence Western Blotting Detection Kit (Amersham Biosciencse, Piscataway, NJ).

### *TP53* mutational analysis

Direct DNA sequencing was performed for exons 4-9 of p53, in which >80% of all mutations were observed [54,55]. Primer sets and reaction conditions were adopted from Lehman *et al* [56]. DNA was amplified by polymerase chain reaction and direct DNA sequencing was performed with the BigDye Sequencing kit (Invitrogen). Electrophoresis and sequence analysis were performed using the ABI PRISM 3100 (Invitrogen).

### *In vivo* tumorigenicity in immunodeficient mice

The study protocol was approved by and performed in accordance with the Committee of the Use of Live Animals in Teaching and Research at the University of Hong Kong. Cells from passage 8 were harvested, washed, and resuspended in hepatocyte culture medium (HCM). 1x10^6^ cells were injected subcutaneously into the right flank of each athymic nude or NOD/SCID mouse (4 weeks old). The mice were examined every week for the development of tumors and tumor-bearing mice were sacrificed when tumor burden exceed 10% of the normal body weight or the body weight loss exceed 20%.

### Immunohistochemical staining

Immunohistochemistry was performed with the Dako Envision Plus System (Dako, Carpinteria, CA) following the manufacturer’s instruction with modifications. Briefly, antigen retrieval was performed by microwave with sections immersed in citrate buffer. Followed by endogenous peroxidase blocking, tissues were stained with rabbit anti-human AFP antibody (Dako) or rabbit Ig (R&D Systems, Minneapolis, MN). The signal was detected by horseradish peroxidase-conjugated secondary antibody and color was developed with diaminobenzidine as the chromogen. The tissue sections were then counterstained with hematoxylin.

### Statistical analyzes

All data were expressed as mean values + standard deviation (SD) from at least three independent experiments. Differences between groups were assessed by the Student’s t test. A probability (p) < 0.05 was considered significantly different. All analyzes were performed using the statistical software GraphPad Prism for Windows, Version 3.00 (GraphPad Software, CA).

## Additional file

Additional file 1:
**Figure S1.** Post-sorting analysis on GEP expression in unsorted Hep3B and freshly isolated GEP^high^, and GEP_low_ subpopulations. **Table S1.** Clinicopathological features of HCC in relation to GEP expression.

## Abbreviations

aCGH: Array-based comparative genomic hybridization
CSC: Cancer stem cell
GEP: Granulin-epithelin precursor
HCC: Hepatocellular carcinoma
STR: Short tandem repeat (STR)
