# Supplementary material for: Establishment and characterization of a novel primary hepatocellular carcinoma cell line with metastatic ability in vivo
Source: Cancer Cell Int. 2014 Oct 9;14:103. doi: 10.1186/s12935-014-0103-y (PMC4209051; doi:10.1186/s12935-014-0103-y)
Supplement: Additional file 1: — Figure S1. Post-sorting analysis on GEP expression in unsorted Hep3B and freshly isolated GEPhigh, and GEPlow subpopulations. Table S1. Clinicopathological features of HCC in relation to GEP expression. [file 12935_2014_103_MOESM1_ESM.doc]

**ADDITIONAL FIGURE LEGEND**

**Additional Figure 1. Post-sorting analysis on GEP expression in unsorted Hep3B and freshly isolated GEP^high^, and GEP_low_ subpopulations.** Cells were sorted based on surface expression of GEP. After cell sorting, cells of each sorted population were collected to assess the cell viability by trypan blue staining and the purity of the sorted subpopulations by flow cytometry using a different anti-GEP antibody (recognizing distinct epitopes compared to the antibodies used for cell sorting). Percentages of GEP+ cells were indicated in the histograms.

**Additional Table 1.** Clinicopathological features of HCC in relation to GEP expression

| **Clinicopathological variables** | **GEP level** | | |
| --- | --- | --- | --- |
|  | **Low** | **High** | ***P* value** |
| **Venous infiltration** |  | | |
| Absence | 9 | 6 | 0.256 |
| Presence | 5 | 8 |  |
| **Tumor size** |  | | |
| Small (≤5cm) | 4 | 5 | 0.686 |
| Large (>5cm) | 10 | 9 |  |
| **Edmondson-Steiner grade** |  | | |
| Well to moderately differentiated | 12 | 10 | 0.044* |
| Poorly to undifferentiated | 0 | 4 |  |
| **Gender** |  | | |
| M | 12 | 9 | 0.190 |
| F | 2 | 5 |  |
| **Age** |  | | |
| Young (≤60) | 11 | 9 | 0.403 |
| Elderly (>60) | 3 | 5 |  |
| **Serum AFP level** |  | | |
| Low (≤20ng/ml) | 7 | 3 | 0.115 |
| High (>20ng/ml) | 7 | 11 |  |
| **HBV association** |  | | |
| Positive for HBsAg | 4 | 2 | 0.357 |
| Negative for HBsAg | 10 | 12 |  |
| * p < 0.05 | | | |
| Abbreviations:  AFP, α-fetoprotein; HBV, hepatitis B virus; HBsAg, hepatitis B surface antigen | | | |
